# Co-Delivery of M2e Virus-Like Particles with Influenza Split Vaccine to the Skin Using Microneedles Enhances the Efficacy of Cross Protection

**DOI:** 10.3390/pharmaceutics11040188

**Published:** 2019-04-18

**Authors:** Min-Chul Kim, Ki-Hye Kim, Jeong Woo Lee, Yu-Na Lee, Hyo-Jick Choi, Yu-Jin Jung, Yu-Jin Kim, Richard W. Compans, Mark R. Prausnitz, Sang-Moo Kang

**Affiliations:** 1 Institute for Biomedical Sciences, Georgia State University, Atlanta, GA 30303, USA; mckim001@gmail.com (M.-C.K.); kkim39@gsu.edu (K.-H.K.); mistybluerain7@gmail.com (Y.-N.L.); yjung8@student.gsu.edu (Y.-J.J.); yujinsm@gmail.com (Y.-J.K.); 2 Komipharm Co., Ltd., Siheung, Gyeonggi-do 15094, Korea; 3 School of Chemical and Biomolecular Engineering, Georgia Institute of Technology, Atlanta, GA 30332, USA; jwlee@gatech.edu (J.W.L.); mark.prausnitz@chbe.gatech.edu (M.R.P.); 4 Animal and Plant Quarantine Agency, Gimcheon, Gyeongsangbukdo 39660, Korea; 5 Department of Chemical and Materials Engineering, University of Alberta, Edmonton, AB T6G 2M9, Canada; hyojick@ualberta.ca; 6 Department of Microbiology and Immunology and Emory Vaccine Center, Emory University School of Medicine, Atlanta, GA 30322, USA; rcompan@emory.edu

**Keywords:** microneedle patch delivery, skin, cross protection, M2e, supplemented vaccines

## Abstract

It is a high priority to develop a simple and effective delivery method for a cross-protective influenza vaccine. We investigated skin immunization by microneedle (MN) patch with human influenza split vaccine and virus-like particles containing heterologous M2 extracellular (M2e) domains (M2e5x virus-like particles (VLP)) as a cross-protective influenza vaccine candidate. Co-delivery of influenza split vaccine and M2e5x VLP to the skin by MN patch was found to confer effective protection against heterosubtypic influenza virus by preventing weight loss and reducing lung viral loads. Compared to intramuscular immunization, MN-based delivery of combined split vaccine and M2e5x VLPs shaped cellular immune responses toward T helper type 1 responses increasing IgG2a isotype antibodies as well as IFN-γ producing cells in mucosal and systemic sites. This study provides evidence that potential immunological and logistic benefits of M2e5x VLP with human influenza split vaccine delivered by MN patch can be used to develop an easy-to-administer cross-protective influenza vaccine.

## 1. Introduction 

Current influenza vaccines do not provide good protection against antigenically distinct strains due to strain-specific protection on the basis of hemagglutinin (HA) subtypes. The influenza virus causes respiratory viral diseases in humans, with significant medical and economic burdens, causing approximately 250,000–500,000 flu-associated deaths annually worldwide [1,2]. In 2009, a novel influenza A (H1N1) virus with distinct antigenic properties emerged and spread globally, resulting in the first 21st century pandemic [3,4,5]. 

To overcome narrow strain-specific protection and to effectively control a pandemic outbreak, new strategies of conferring broadly cross-protective immunity against antigenically different influenza strains are under development [6,7]. Due to the significant genetic and antigenic differences between influenza A and B viruses, a single vaccine that protects against both types may not be realistic. A feasible approach is to develop an improved vaccination that is more broadly cross protective than the currently licensed influenza vaccines without the need for updating every year or it should have improved efficacy against heterosubtypic influenza viral infection. 

In contrast to the variable critical surface antigens of HA and neuraminidase (NA) surface antigens, the ion-channel protein M2, representing a candidate antigen for universal influenza A vaccine, has an extracellular domain of 24 amino acids (M2e) with a conserved molecular target among human influenza A strains [8]. Our recent study reported a molecular construct with a repeat of heterologous M2e peptide sequences (M2e5x) expressed in a membrane-anchored form and was presented on enveloped virus-like particles (VLPs) as a universal influenza A vaccine candidate (M2e5x VLPs) [9]. 

Microneedles (MNs) are micron-scale, solid needles that can be coated with dried vaccines and applied to the skin as a patch in a simple and painless manner. MN patch delivery of vaccines would enhance vaccination coverage by possible self-vaccination or by simply instructed personnel. Enhanced immunogenicity was observed by vaccination in the skin [10] and the feasibility of easy skin vaccination via MNs was further reported in recent studies [11,12,13], including in human clinical trials [14,15]. Recent approaches were demonstrated to induce heterosubtypic cross protection by influenza vaccination using MNs although non-neutralizing M2e immunity alone would not be sufficient for conferring sufficient protection against circulating and antigenically different strains [16,17]. 

In an effort toward achieving more effective vaccine delivery of a broadly protective vaccine that provides protection against circulating and antigenically different multiple strains of influenza, we hypothesized that the MN-based co-delivery of licensed influenza split vaccine and M2e5x VLPs as a conserved molecular antigen would overcome strain-specific protection of a current vaccination strategy. In this study, we tested MN patches to co-immunize with licensed influenza split vaccine and M2e5x VLP. The present study investigates humoral and cellular immunogenicity as well as heterosubtypic protection of an influenza split vaccine co-immunized with M2e5x VLPs through skin immunization using MN delivery.

## 2. Materials and Methods

### 2.1. Viruses, Influenza M2e5x VLPs, and Human Influenza Split Vaccine

Using embryonated chicken egg substrates, we propagated influenza viruses including A/PR/8/34 (H1N1, A/PR8), A/California/04/2009 (H1N1, A/Cal), A/Philippines/2/1982 (H3N2 virus, A/Phil), and A/Mandarin duck (rgH5N1, avian rgH5N1 containing HA with polybasic residues removed, NA, and M (containing avian M2e) genes from A/Mandarin duck/Korea/PSC24-24/2010, and the remaining backbone genes from A/PR8 virus). A/PR8 (H1N1), A/Cal (H1N1), A/Phil (H3N2), and A/Mandarin duck (rgH5N1) viruses were inactivated by treating formalin as previously described [18]. Human influenza split vaccine derived from the 2009 pandemic strain of A/California/07/2009 (H1N1) virus was generously provided by Green Cross (Yongin-si, Korea) or Seqirus (Maidenhead, United Kingdom). M2e5x VLPs containing tandem repeat of human, swine, and avian influenza virus M2e epitopes were produced in insect cells using the recombinant baculovirus expression system as previously described [9]. 

### 2.2. Immunogold Cryogenic-Transmission Electron Microscopy of M2e5x VLPs

M2e5x VLP vaccine preparations were examined to determine particle morphology and sizes by cryogenic-transmission electron microscopy (cryo-TEM, JEOL 2200 FS; JEOL USA, Peabody, MA, USA). M2e5x VLP cryo-TEM samples were prepared by plunge-freezing thin aqueous films as described in a previous study [17]. In short, M2e5x VLP samples (1 μg) were coated onto formvar/carbon-coated copper grids (Electron Microscopy Sciences, Fort Washington, PA, USA). To stain M2e epitopes on vaccine particles, M2e monoclonal mouse antibody (14C2, Abcam) was incubated on the grid for 1 h. Then, secondary 6 nm gold-conjugated anti-mouse antibody (Abcam) was applied to the grid and incubated for 15 min. After washing, 1.5% phosphotungstic acid (pH 7.0) was briefly applied to negatively stain the grids and the images of M2e5x VLP cryo-TEM were captured.

### 2.3. Preparation of MN Patches

An MN patch to administer vaccine was prepared by fabricating solid MN arrays and coating vaccine antigens on the surface of the MNs as described previously [17,19]. A separate patch with an array of five MNs coated with 2 µg of influenza M2e5x VLPs or 0.3 µg of influenza split vaccine was used to vaccinate animals. Mock vaccination was carried out using placebo-MNs without antigens.

### 2.4. Immunization and Challenge 

Female inbred BALB/c mice (Charles River) aged 6 to 8 weeks were used. Groups of mice (12 mice per group) were immunized with separate MN arrays coated with M2e5x VLP (2 µg of total proteins) and/or split vaccine (0.3 µg of HA) for skin delivery in each mouse. Each array of vaccine-coated MNs was inserted into the skin and held for 10 min for release of the vaccine antigen from the coated MNs. Co-immunization with split vaccine MN and M2e5x VLP MN was performed on the skin of mice via MN delivery (S-M2e5x MN) at a 4 week interval. For an intramuscular (IM) control group, mice were intramuscularly immunized with M2e5x VLP on one leg and split vaccine on another leg (S-M2e5x IM) or M2e5x VLP (2 µg) alone. At 6 weeks after boost, mice were intranasally challenged with a lethal dose of A/Philippines/2/82 (4 × 50% lethal dose [LD50]), and body weight changes and survival rates daily monitored. All animal experiments and the humane care of the animals were followed by the protocol (A14025) approved by the Georgia State University IACUC review board. 

### 2.5. Antibody Responses and Hemagglutinin Inhibition (HAI) Assay

M2e or virus-specific antibody responses were determined using human, swine, and avian M2e peptide antigens or inactivated viruses, A/PR8 (H1N1), A/California, A/Philippines (H3N2), and A/Mandarin duck (rgH5N1), as ELISA coating antigen (2 µg/mL) as previously described [18]. Hemagglutination (HA) inhibition (HAI) activity titers were determined using 4 HA units of homologous, heterologous, and heterosubtypic influenza viruses as described [20,21]. 

### 2.6. Analysis of Antibodies in Bronchoalveolar Lavages after Challenge

Bronchoalveolar lavage fluids (BALF) were prepared from mice (*N* = 4) by infusing 1 mL of phosphate-buffered saline (PBS) into lungs at day 4 after challenge for the analysis of antibody responses and cytokines as described [9]. 

### 2.7. Inflammatory Cytokine and Lung Viral Titer 

The Ready-Set-Go cytokine kit (eBioscience, San Diego, CA, USA) was used to determine cytokine interleukin-6 (IL-6) in BALF. A method of mechanical tissue grinding was utilized to prepare lung extracts in 1.5 mL phosphate-buffered saline (PBS) and viral titers were determined. Embryonated chicken eggs were inoculated with diluted lung extracts to determine influenza viral titers by measuring HA activity in allantoic fluids as described [22]. 

### 2.8. Determination of T Cell Responses 

T cell responses in lung and spleen cells were determined at day 4 post challenge by an ELISpot assay using interferon (IFN)-γ specific capture antibodies as described [23,24,25]. Concisely, spleen (0.5 × 10^6^ cells per well) or lung cells (0.2 × 10^6^ cells per well) were cultured for 36 h on the capture antibody-coated ELISpot plates containing inactivated influenza A/California (pdmH1N1), inactivated A/Philippines (H3N2), and M2e peptide as an antigenic stimulator (2 µg/mL). An ELISpot reader (BioSys, Miami, FL, USA) was utilized to count the spots of IFN-γ secreting T cells after 36 h incubation.

### 2.9. Cross-Protective Efficacy Test of Immune Sera 

To test cross-protective efficacy to more heterologous or heterosubtypic influenza viruses, immune sera were collected at 4 weeks after boost inoculation. In brief, sera were heat-inactivated at 56 °C for 30 min and the serum samples were mixed with the same volume of 6 × LD_50_ of influenza viruses—A/PR8 (heterologous H1N1) and A/Mandarin duck (heterosubtypic avian rgH5N1)—and incubated for 1 h at room temperature. The mixture was intranasally administered to naive mice (*N* = 3, BALB/c) and body weight and survival rates were daily monitored for 14 days.

### 2.10. Statistical Analysis

To determine the statistical significance, a two-tailed Student’s *t* test was used when comparing two different conditions. The statistical analysis was performed by one-way analysis of variance statistics (ANOVA) with a Tukey multiple-comparison test in GraphPad Prism for comparing multiple groups. Kaplan survival statistical analysis was performed for survival-curves. A *p*-value less than 0.05 was considered to be statistically significant. 

## 3. Results

### 3.1. M2e5x VLPs Present M2e Epitopes on the Particle Surfaces 

**Figure 1:**
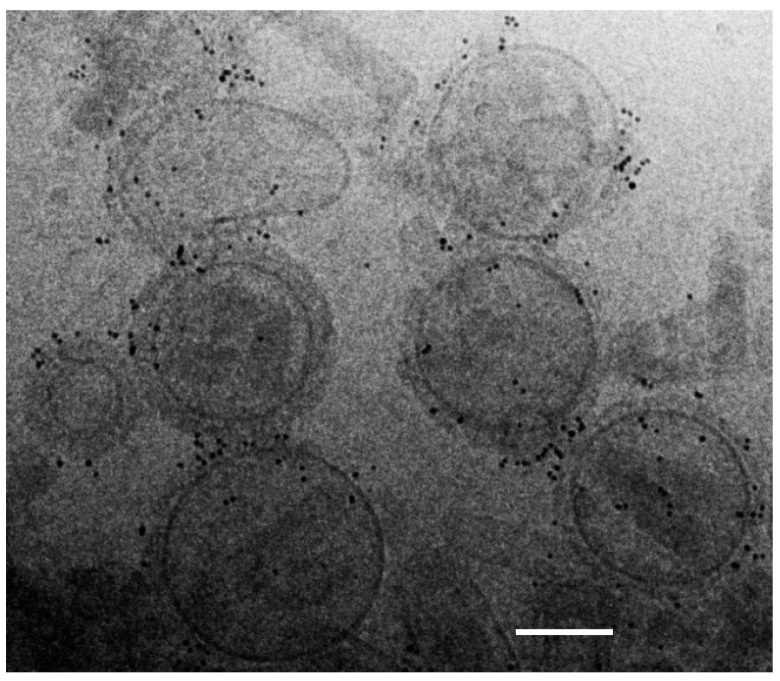
Cryogenic-transmission electron microscopy (cryo-TEM) of M2e5x virus-like particles (VLPs). Morphology and size of M2e5x VLPs were examined using cryogenic-transmission electron microscope (cryo-TEM). Mouse anti-M2e antibody (14C2) and 6 nm gold-conjugated goat anti-mouse antibodies were used as the primary and secondary antibodies to probe the M2e epitopes on VLPs. Bar scale: 100 nm.

We examined morphological integrity and M2e epitope presentation of M2e5x VLP vaccines using cryogenic-transmission electron microscopy (EM). Morphology of spherical particles with a range of 100 to 160 nm sizes was observed by EM, as shown in Figure 1. Presentation of M2e epitopes on M2e5x VLPs was probed under the EM after reaction with M2e specific monoclonal 14C2 antibody and gold particle-conjugated secondary antibody. Numerous gold particle spots on the surfaces of M2e5x VLPs were clearly visible, suggesting that M2e epitopes are sufficiently well exposed to access by M2e antibodies, as shown in Figure 1. In contrast, the influenza virus did not show such gold particle spots on the surfaces (data not shown), probably due to the inaccessible exposure or insufficient levels on the virion particles. These results suggest that M2e5x VLPs maintain virus-like structural and morphological integrity, presenting M2e epitopes accessible to antibodies on their surfaces.

### 3.2. M2e5x VLP Co-Immunization with Split Vaccine in the Skin by MN Patch Enhances M2-Specific Antibody Responses

**Figure 2:**
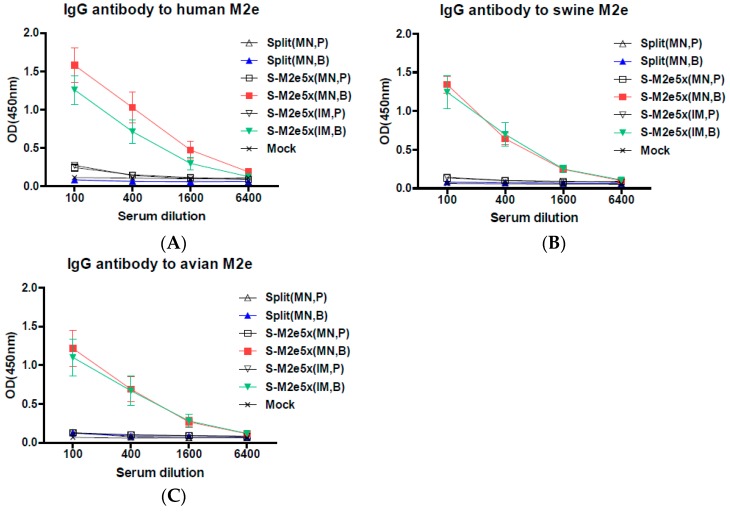
M2e5x VLP supplemented vaccination induces M2e-specific antibodies. BALB/c mice were microneedle (MN) immunized on the skin with split vaccine alone (Split MN, *N* = 12), co-immunized with split vaccine and M2e5x VLP via separate MN delivery to the skin (S-M2e5x MN, *N* = 12), or immunized with split vaccine M2e5x VLP on the different legs by intramuscular (IM) route (S-M2e5x IM, *N* = 12). Sera were collected 3 weeks after prime (P) and boost (B) vaccination. The IgG level was detected using M2e peptides as an ELISA coating antigen. (**A**) IgG antibody responses to human M2e peptide. (**B**) IgG antibody responses to swine M2e peptide. (**C**) Comparison of IgG antibody responses to avian M2e peptide. Error bars indicate mean ± SEM.

We investigated whether MN delivery to the skin by supplementing M2e5x VLPs as a source of conserved antigenic target to the strain-specific influenza split vaccine would enhance M2e immune responses and subsequently the breadth of cross protection against influenza A viruses. Groups of mice were immunized with 2009 H1N1 split vaccine (Split MN) alone, split MN vaccine plus M2e5x VLP MN (S-M2e5x MN) by MN delivery to the skin, and separate split vaccine plus separate M2e5x VLPs (S-M2e5x IM) by intramuscular (IM) injection, as shown in Figure 2. Human M2e-specific antibodies were induced at substantially high levels in the S-M2e5x VLP MN group and further increased by approximately 60-fold after boost, as shown in Figure 2A. The split MN vaccine alone group did not show M2e-specific antibody responses, as shown in Figure 2A. The M2 ectodomain sequence has few residue variations among human, swine, and avian influenza A viruses. Next, we determined if S-M2e5x VLP immune sera would be reactive with swine or avian influenza A virus M2e antigens, as shown in Figure 2B,C. The highest levels of IgG antibodies specific for a swine or avian M2e antigen were observed in S-M2e5x MN immune sera. When different routes of vaccination were compared, similar levels of M2e-specific IgG antibodies were induced in MN skin and IM boost immune sera from S-M2e5x MN co-immunization, as shown in Figure 2A–C. In summary, S-M2e5x MN skin vaccination induces IgG antibodies specific for human, swine, and avian M2e antigens, at comparable levels to those of IM injection with S-M2e5x vaccines. 

### 3.3. M2e5x VLP Co-Immunization with Split Vaccine Increases the Ratio of IgG2a/IgG1 Isotypes and Heterosubtypic Virus-Specific IgG Antibody Responses

**Figure 3:**
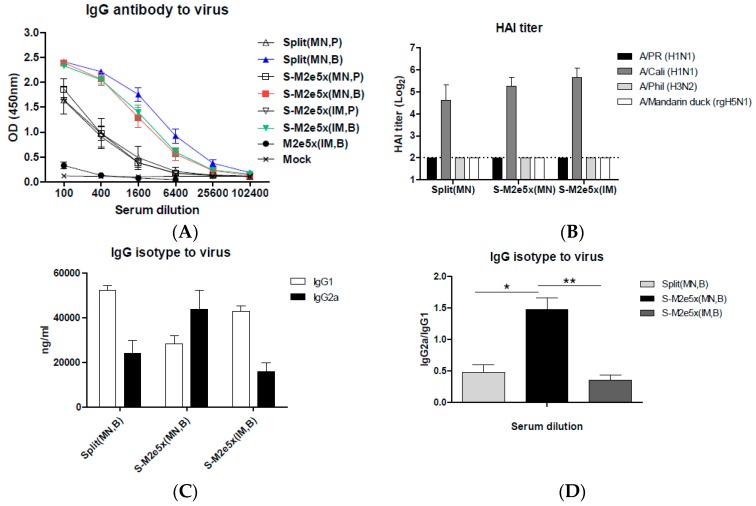
M2e5x VLPs co-immunized MN vaccination changed IgG isotype to viral antigens. BALB/c mice (*N* = 12) were immunized with split vaccine alone (Split MN) or split vaccine supplemented with M2e5x VLPs (S-M2e5x MN). IM control groups (*N* = 12) include S-M2e5x (IM) and M2e5x (IM). (**A**) IgG antibody responses to influenza A/Cal virus. Sera were collected 3 weeks after prime (P) and boost (B) vaccination. The IgG level was detected using influenza A viruses, A/California/04/09 (A/Cal, H1N1) as an ELISA coating antigen. (**B**) Hemagglutinin inhibition titers of immune sera. Hemagglutinin inhibition (HAI) titers were analyzed with boost sera as a measure of functional antibody responses against A/California/04/09 (H1N1), A/PR/8/34 (H1N1), A/Philippines/2/82 (H3N2), and A/Mandarin duck/PSC24-24/2010 (rgH5N1) influenza viruses. The dotted line denotes the limit of HAI detection. (**C**) Comparison of IgG isotype antibody responses to influenza A/Cali virus. (**D**) Ratio of IgG isotype antibody (IgG2a/IgG1). Error bars indicate mean ± SEM. For statistical analysis, one-way ANOVA and Tukey’s post-multiple comparison test were performed. *; *p* < 0.05, **; *p* < 0.001 between the indicated groups.

Levels of antibody responses specific for influenza A/California (H1N1) virus were similarly high in the S-M2e5x MN group (Split MN + M2e5x MN) and the split vaccine alone group (Split MN), as shown in Figure 3A. In contrast, the M2e5x VLP alone group showed low levels of IgG antibodies specific for virus, as shown in Figure 3A. HAI titers against homologous virus (A/California, H1N1) as a measure of virus neutralizing functional antibodies were detected at high levels in immune sera of the S-M2e5x MN, S-M2e5x IM, and split MN groups, as shown in Figure 3B. However, serum antibodies from all immunized groups did not show HAI activity to heterologous A/PR8 virus or heterosubtypic influenza viruses, A/Phil (H3N2) or A/Mandarin duck (rgH5N1), as shown in Figure 3B. 

To further understand types of immune responses to influenza virus, we analyzed IgG isotypes (IgG1 and IgG2a) of serum antibodies after boost vaccination, as shown in Figure 3C,D. Split MN and S-M2e5x IM route showed higher levels of virus-specific IgG1 antibodies than those of IgG2a isotype antibodies. In contrast, higher levels of IgG2a than those of IgG1 antibody were induced in the S-M2e5x MN co-immunized group by skin route, as shown in Figure 3C. As a result, approximately 3-fold higher levels of IgG2a/IgG1 ratios were induced in the S-M2e5x MN group by skin route comparing to Split MN and S-M2e5x IM, as shown in Figure 3D. These results provide evidence that T helper type 1 (Th1) immune responses to viral antigen can be effectively induced by co-delivery of split and M2e5x VLP vaccines to the skin via MNs. 

**Figure 4:**
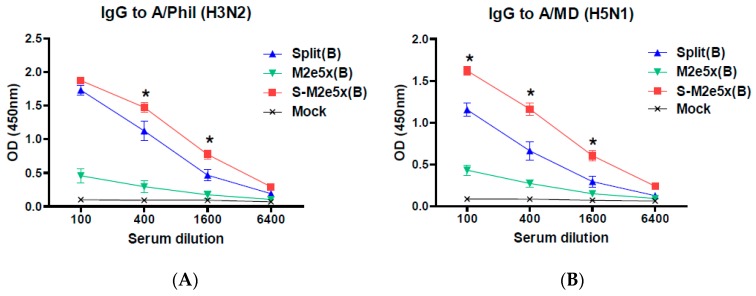
M2e5x VLP supplemented influenza split vaccination induces enhanced IgG antibody responses to different subtype viral antigens. BALB/c mice (*N* = 6) were immunized with A/Cal split vaccine (Split), M2e5x VLP alone (M2e5x), or split vaccine supplemented with M2e5x VLPs (S-M2e5x). Sera collected 3 weeks after boost (B) vaccination were used to determine IgG antibody responses to different subtype viral antigens. (**A**) IgG antibody responses to influenza A/Phil (A/Philippines/2/82, H3N2). (**B**) IgG antibody responses to influenza rgH5N1 (A/PR8 reassortant containing HA, NA, and M from A/Mandarin duck/PSC24-24/2010). One-way ANOVA and Tukey’s post-multiple comparison test were performed. *; *p* < 0.05 between the split and S-M2e5x groups.

Next, we determined the levels of IgG antibodies recognizing heterosubtypic viruses of A/Philippines (H3N2) and rgH5N1 (A/Mandarin with avian M2e) in boost immune sera, as shown in Figure 4. The M2e5x VLP alone group induced low levels of IgG antibodies specific for A/Philippines, as shown in Figure 4A, and A/Mandarin viruses, as shown in Figure 4B. Importantly, the S-M2e5x group showed higher levels of IgG antibodies binding to heterosubtypic viruses than those in the split vaccine alone group, as shown in Figure 4. Influenza split vaccination induces strain-specific virus neutralizing antibodies at similar levels in all groups with split vaccine, as shown in Figure 3B. Therefore, virus strain-specific neutralizing titers and heterosubtypic virus binding IgG levels are not well correlated. 

### 3.4. M2e5x VLPs Co-Immunized with Split Vaccines Provide Enhanced Cross Protection 

**Figure 5:**
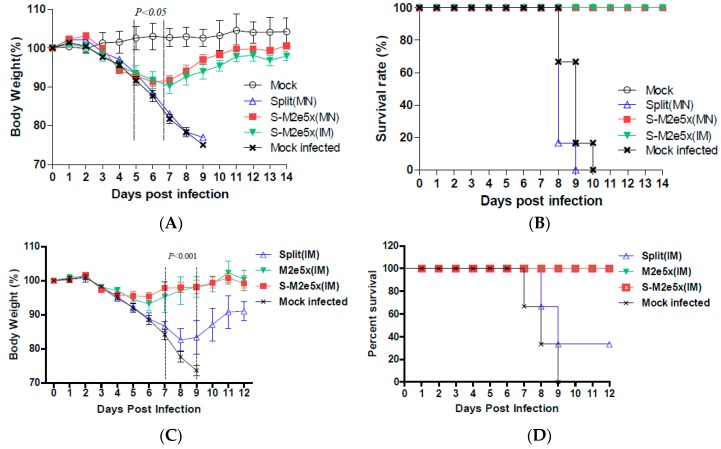
Improved efficacy of cross protection by supplemented S-M2e5x (MN, IM) and M2e5x VLP vaccine. Groups of mice (*N = 6*) that were immunized on the skin via MN (Split MN, S-M2e5x MN) or IM injection (S-M2e5x IM) were intranasally challenged with a lethal dose (5 × LD_50_) of influenza viruses, A/Philippines/2/82 (H3N2), 6 weeks after boost vaccination. (**A**) Average body weight changes after challenge with A/Philippines (H3N2). (**B**) Survival rates after challenge with A/Philippines (H3N2). Body weight and survival rates were monitored for 14 days. (**C**,**D**) In an independent set of experiments to test M2e5x VLP alone and supplemented S-M2e5x vaccines in comparison with split vaccine, groups of mice (*N = 6*) that were IM immunized with M2e5x VLP (2.5 µg), Split (0.3 HA µg), or S-M2e5x [Split (0.3 HA µg) + M2e5x VLP (2.5 µg)], and intranasally challenged with a lethal dose (3 × LD_50_) of A/Philippines/2/82 (H3N2) virus at 6 weeks after boost. (**C**) Average body weight changes. (**D**) Survival rates. One-way ANOVA and Tukey’s post-multiple comparison test were performed. P value indicates a significant difference among the S-M2e5x (MN, IM) or M2e5x (IM) and split MN or IM groups. Data represent mean ± SEM.

To compare the efficacy of M2e5x VLP co-immunized with split vaccine in conferring cross protection against lethal challenge, groups of mice were immunized via MN skin delivery and intranasally challenged with a lethal dose (4 × LD_50_) of heterosubtypic influenza virus, A/Philippines (H3N2), at 6 weeks after boost, as shown in Figure 5A,B. Mice that were vaccinated with the M2e5x VLP and split vaccine showed a slight loss of 8% in body weight post challenge, resulting in 100% protection (S-M2e5x MN), as shown in Figure 5B, similar to that of the S-M2e5x VLP IM group. In contrast, mice vaccinated with the 2009 H1N1 split vaccine alone showed a significant weight loss (~25%), reaching the end points, resulting in 0% survival rates. All naïve mice also lost over 25% in body weight and had to be euthanized. These results demonstrate that M2e5x VLP co-immunization using MNs with split vaccine is superior to split vaccine alone MN in conferring cross protection. 

To further determine the roles of M2e immunity in conferring cross protection, the efficacy of the M2e5x VLP alone group was compared with that of the S-M2e5x group in an independent experimental set, as shown in Figure 5C,D. The M2e5x VLP supplemented S-M2e5x group displayed a similar pattern of body weight compared to that of the M2e5x VLP group. As expected, both M2e5x VLP and S-M2e5x VLP immunized mice showed better cross protection than the mice immunized with split vaccine alone, as shown in Figure 5C,D.

### 3.5. S-M2e5x VLP MN Co-Immunization Contributes to Inducing M2e Mucosal Antibodies 

**Figure 6:**
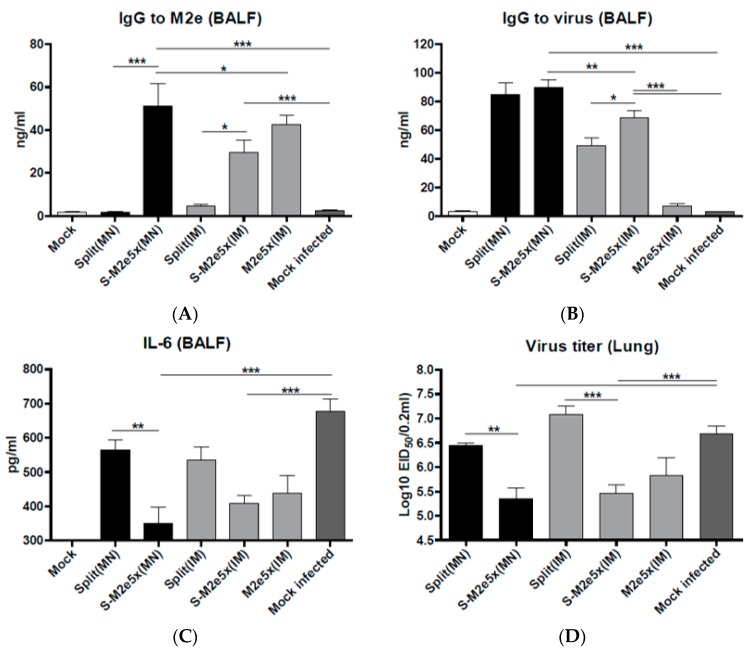
M2e-specific IgG antibodies, inflammatory IL-6 cytokine, and lung viral titers. Levels of IgG antibodies and cytokines in bronchoalveolar lavage fluids (BALF) samples collected from mice were determined at day 4 post-challenge (*N* = 4) at 8 weeks after boost vaccination. (**A**) IgG antibody level to A/California/04/09 (H1N1) virus. (**B**) M2e specific IgG antibody responses to M2e peptide. IgG antibody responses were determined by ELISA using inactivated influenza virus (A/Cal) or human type M2e peptide as a coating antigen (4 ug/mL). (**C**) Inflammatory IL-6 cytokine in BALF. IL-6 was determined by a cytokine ELISA (*N* = 4). (**D**) Lung viral titers. Lung viral titers were determined by an egg inoculation assay day 4 post challenge (*N* = 4). Error bars indicate mean ± SEM. BALF, bronchoalveolar lavage fluid; pg, picogram. MN: skin immunization by MN patch, IM: intramuscular needle injection immunization. One-way ANOVA and Tukey’s post-multiple comparison test were performed. *; *p* < 0.05, **; *p* < 0.01, ***; *p* < 0.001 between the marked groups.

M2e- or virus-specific IgG antibody responses in BALF were determined at day 4 after viral challenge with the H3N2 virus, as shown in Figure 6A,B. Significantly higher levels of IgG specific for M2e were observed in the airway BALF in the co-immunization S-M2e5x (MN, IM) or M2e5x VLP groups, as shown in Figure 6A. Importantly, MN delivery of S-M2e5x was more effective in inducing M2e and virus specific antibodies in mucosal BALF than IM immunization with S-M2e5x, as shown in Figure 6A,B. Meanwhile IgG antibody responses specific for virus were observed to be at significantly higher levels in both the split vaccine alone and co-immunized S-M2e5x either MN or IM, as shown in Figure 6B. Therefore, these results suggest that M2e5x VLP co-immunization with split vaccine using MNs can effectively induce M2e specific antibodies in respiratory mucosal sites.

### 3.6. S-M2e5x VLP Co-Immunization Lowers Inflammatory Cytokine Levels and Lung Viral Titers

Excess production of inflammatory cytokines due to influenza virus infection may be associated with severe disease. Cytokine interleukin (IL)-6 in BALF from mice of split MN or S-M2e5x VLP MN vaccine after challenge was determined, as shown in Figure 6C. Highest levels of IL-6 were observed in BALF from naïve mice, indicating lung inflammatory responses. The level of IL-6 in BALF from S-M2e5x MN or IM co-immunized mice was lower than that of split alone MN or IM immunized mice. To better assess the protective efficacy against A/Philippines (H3N2), lung viral titers were determined at day 4 post challenge. The group of S-M2e5x MN or IM vaccine showed approximately 10-fold lower lung viral titers (A/Philippines, H3N2) compared to those in both split vaccine and naïve-challenge control groups, as shown in Figure 6D. Therefore, M2e specific immune responses in systemic and respiratory mucosal BALF sites may effectively contribute to controlling heterosubtypic virus replication and thus reduce inflammatory IL-6 cytokine production as a result of S-M2e5x MN skin or IM co-immunization. 

### 3.7. S-M2e5x VLP Co-Immunization Enhances IFN- γ Secreting T Cell Responses

**Figure 7:**
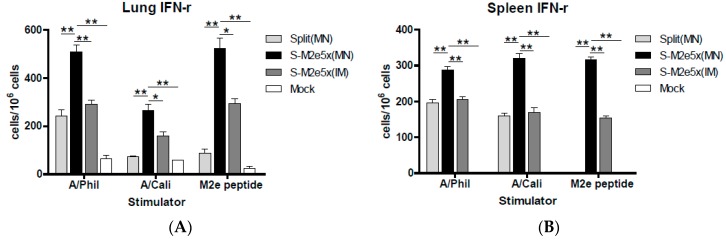
Systemic IFN-γ secreting cell spots after MN immunization upon heterosubtypic challenge. Splenocytes and lung cells were isolated from mice at day 4 post-challenge (*N* = 4) with A/Philippines (H3N2). IFN-γ secreting cells were detected in the presence of A/Philippines/2/82 (H3N2) virus, A/California/04/09 (H1N1) virus, or M2e peptide as a stimulator (2 µg/mL). (**A**) IFN-γ secreting lung cells. (**B**) IFN-γ secreting splenocytes. IFN-γ cytokine-producing cells were counted by ELISpot reader. Data represent mean ± SEM. IFN, interferon. MN: skin immunization by MN patch, IM: intramuscular needle injection immunization. One-way ANOVA and Tukey’s post-multiple comparison test were performed. *; *p <* 0.05, **; *p <* 0.01 between the marked groups.

M2e peptide has T-cell epitopes in mice [26,27] and the M2 protein specific human CD4 and CD8 cytotoxic T cell epitopes have been described in previous studies [28,29]. Cells from spleen or lung samples were collected at day 4 post-challenge to determine IFN-γ producing cellular responses, as shown in Figure 7A,B. The group of mice that was immunized with S-M2e5x MN or IM vaccine showed significantly higher levels of IFN-γ secreting cell spots compared to those of split vaccine alone group in both spleen and lung cells in response to stimulation with A/Philippines/2/82 (H3N2), A/California/04/2009 (H1N1), M2e peptide, or M2e5x VLPs. Also, IFN-γ secreting cell responses in lungs were found to be higher in the S-M2e5x group than M2e5x alone group (data not shown). No or low levels of IFN-γ secreting spleen or lung cells were observed in naïve mice after challenge. These results provide evidence that M2e5x VLP co-immunization with split vaccine using MNs effectively induces INF-γ secreting T cells in response to influenza virus or M2e antigens.

### 3.8. S-M2e5x MN Co-Immune Sera Confer Enhanced Cross Protection.

**Figure 8:**
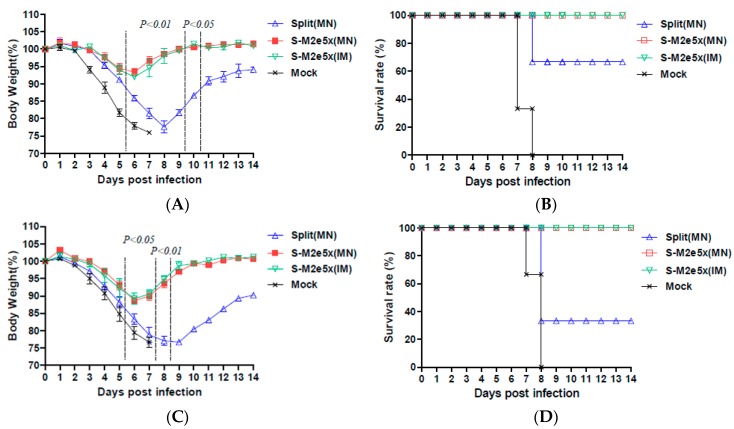
Immune sera from mice with split and M2e5x VLP vaccines together confer improved cross protection. Naïve mice (*N* = 3) were intranasally infected with 3 × LD_50_ of influenza virus mixed with immune sera. (**A**) Body weight changes and (**B**) survival rates in naïve mice after infection with a mixture of A/PR8 (heterologous H1N1) virus and immune or mock sera. (**C**) Body weight changes and (**D**) survival rates in naïve mice after infection with a mixture of A/Mandarin duck (rgH5N1) virus and immune or mock sera. For statistical analysis, one-way ANOVA and Tukey’s post-multiple comparison test were performed. P values indicate significant differences between the immune sera of split MN or S-M2e5x MN as marked in the graph. Data represent mean ± SEM. Split MN; immune sera of split MN mice, S-M2e5x MN; co-immune sera of S-M2e5x MN mice, S-M2e5x IM; immune sera of S-M2e5x IM mice.

A tailored passive transfer was employed to determine the roles of M2e vaccine immune sera in conferring cross protection as described [9,24,30,31]. Naïve mice that were intranasally inoculated with M2e immune sera (S-M2e5x MN, S-M2e5x IM) mixed with pathogenic virus were well protected against A/PR8 (H1N1), as shown in Figure 8A,B, or A/Mandarin duck (rgH5N1), as shown in Figure 8C,D, as evidenced by less than 5% or 10% weight loss, respectively, and then rapid recovery, resulting in 100% survival. No difference was found between immune sera from the S-M2e5x MN and S-M2e5x IM groups, whereas naïve mice that received immune sera of split MN vaccine showed approximately more than 20% weight loss indicating severe illness, resulting in 66% (A/PR8) and 33% (A/Mandarin duck) survival rates. Naïve serum-treated mice exhibited most severe weight loss, resulting in no survival. Therefore, in summary, IgG antibodies induced by S-M2e5x MN and S-M2e5x IM contribute to conferring cross protection against antigenically different H1, H3, and H5 subtype influenza A viruses at higher efficacy than those by split vaccine alone. Under a low dose of antigenically different virus, split vaccine alone immune sera provided weak survival cross protective roles accompanying severe weight loss. Therefore, both antibodies against M2e and split vaccine are likely contributing to cross protection where M2e antibodies might play a major role.

## 4. Discussion

Immunity from current influenza vaccination is targeted to distinct HA proteins. However, it is difficult to anticipate the next seasonal and pandemic strain because influenza viruses are continuously evolving by mutations in the highly variable HA proteins. Most people are susceptible to antigenically drifted or pandemic strains despite existing HA immunity. The emergence of the 2009 pdmH1N1 and recent H3N2 variants are examples of the generation of a new strain with distinct antigenic properties [4,5,32,33,34]. It would be an ideal goal to develop a highly effective stand-alone universal influenza vaccine. In addition to universal vaccine approaches, developing simple and effective vaccination methods as an alternative to conventional needle-and-syringe injection has been also gaining attention. MN patches have been developed as an effective tool for delivering vaccine antigens or drugs to the skin [11,12] because of patient-friendly properties such as simple and painless administration.

Developing a universal influenza vaccine might be achievable although there are antigenic differences on the surface HA and NA proteins and relatively low immunogenicity of conserved antigenic targets. Previous studies on MN influenza immunizations in mice have investigated vaccine immunogenicity and efficacy against homologous and heterologous viruses [35,36,37,38]. Wang et al. reported that MN delivery of Toll-like receptor 5 agonist bacterial flagellin adjuvant conjugates with homologous M2e tandem repeat had a potential to protect against heterosubtypic viral infection with significant weight loss of over 16% [39]. In our previous study, M2e5x VLP MN alone vaccine also had potential to protect against viral infection [17]. However, non-neutralizing M2e immunity alone would not provide sufficient protection compared to HA vaccines against homologous viruses [40,41]. An alternative approach is to improve the capacity of the HA strain specific current influenza split vaccine to confer cross protection. Intramuscular co-immunization with human influenza split vaccine and M2e5x VLP was able to overcome strain-specificity by conferring broader cross protection [17]. Also, subsequent immunization with M2e5x VLP together with HA specific pre-existing immunity elicited cross protection against later heterosubtypic challenge [31]. Recently, it was reported that boost vaccination with a dissolving microneedle patch encapsulating M2e vaccine broadened the protective efficacy of conventional influenza vaccines although weight losses ranging 10–20% were observed depending on the challenge strains [16]. In this study, we investigated the cross-protective efficacy of co-immunization with split vaccine and M2e5x VLP via MN delivery to the skin. MN co-immunization with S-M2e5x MN effectively induced M2e-specific humoral and cellular immune responses. Notably, mice immunized with S-M2e5x exhibited high levels of IgG antibodies binding to heterosubtypic viruses and enhanced cross protection against antigenically distinct heterologous and heterosubtypic influenza viruses. Therefore, MN co-vaccination with M2e5x VLP and influenza split vaccine can improve the efficacy of cross protection. More importantly, M2e5x VLPs vaccines would not have seasonality and could be administered as a supplemented vaccine during seasonal vaccination, prior to or after seasonal vaccination.

Protective mechanisms mediated by M2e antibodies have not been well defined because anti-M2e antibodies do not directly neutralize the virus [24,42,43]. In our previous study, M2e-specific antibody was reactive to M2-expressing or virus-infected MDCK cells [21,31], suggesting that M2e-binding antibody on the infected cells can mediate opsonophagocytosis. In vivo cross protection by M2e antibodies was demonstrated to require the engagement of Fc receptors [21,44,45] and possibly alveolar macrophages [24]. It is likely that M2e antibodies contribute to broad protection against influenza virus via Fc-mediated effector mechanisms, such as antibody dependent cellular cytotoxicity [46] or antibody dependent cellular phagocytosis of immune complexes [47].

In addition to M2e antibodies, a profile of IgG isotypes may differentially contribute to antiviral immunity. IgG1 isotype antibodies are likely to be involved in neutralizing homologous virus [48]. Meanwhile, IgG2a isotype antibodies might play a role in assisting the clearance of virus-infected host cells [48]. Regarding the protective roles of different isotypes, M2e specific IgG1 monoclonal antibody (mAb) required Fcγ receptor III whereas M2e specific IgG2a required the presence of Fcγ receptor IV [44]. In our previous studies, inclusion of M2e5x VLP in the split vaccination or subsequent immunization under virus-specific pre-existing immunity was shown to modulate the pattern of IgG isotypes toward a Th1 type [21,31]. In this study, it is interesting to note that M2e5x VLP co-immunization resulted in an increase in IgG2a antibody levels, whereas the split MN vaccine or IM immunization with separate split and M2e5x VLP (2.5 µg) vaccines on different legs showed IgG1 isotype dominant antibody responses. It is speculated that the cells taking up the antigens and antigen-presenting cells would be different after MN delivery compared to IM injection. MN vaccine delivery to the skin was shown to induce cytokines and to recruit neutrophils, monocytes, and dendritic cells at the site of immunization, and prolonged antigen deposition, resulting in the migration of antigen-bearing antigen-presenting cells such as dendritic cells to the draining lymph nodes [49]. This study suggests that the difference of IgG2a/IgG1 ratio does not affect cross protection significantly. In our previous study, IM co-immunization of mixed M2e5x VLP (10 µg) and split vaccine resulted in a significant increase in IgG2a antibody levels [21], which suggest that the M2e5x VLP vaccine would have a role in modulating immune responses to a co-immunized vaccine. It would therefore be desirable to do further study using MN vaccine mixed with human split vaccine and M2e5x VLP vaccine.

In summary, this study demonstrated heterosubtypic protection via MN delivery of M2e5x VLP and licensed human split vaccines. MN co-immunization with M2e5x VLP and split vaccine conferred 100% cross protection against challenge with antigenically different heterosubtypic influenza viruses, whereas vaccination using split vaccine alone provides minimal cross protection. The results also demonstrated that MN co-immunization with M2e5x VLP and split vaccines could have a dual role of inducing M2e immunity and modulating host immune responses to vaccine toward the Th1 responses. In conclusion, MN-based co-immunization with M2e5x VLP and a current platform of human influenza vaccines has promising potential for inducing cross-protective immunity. Overall, this study shows that MN-based delivery of influenza split vaccines and M2e5x VLP vaccines together could be an advantageous approach to enable the protection against heterosubtypic influenza viral infection.
